# Altered Antibody Profiles against Common Infectious Agents in Chronic Disease

**DOI:** 10.1371/journal.pone.0081635

**Published:** 2013-12-02

**Authors:** Peter D. Burbelo, Kathryn H. Ching, Caryn G. Morse, Ilias Alevizos, Ahmad Bayat, Jeffrey I. Cohen, Mir A. Ali, Amit Kapoor, Sarah K. Browne, Steven M. Holland, Joseph A. Kovacs, Michael J. Iadarola

**Affiliations:** 1 Clinical Dental Research Core, National Institute of Dental and Craniofacial Research, National Institutes of Health, Bethesda, Maryland, United States of America; 2 Western Regional Research Center, U.S. Department of Agriculture, Albany, California, United States of America; 3 Critical Care Medicine Department, Clinical Center, National Institutes of Health, Bethesda, Maryland, United States of America; 4 Sjögren Syndrome Clinic, Molecular Physiology and Therapeutics Branch, National Institute of Dental and Craniofacial Research, National Institutes of Health, Bethesda, Maryland, United States of America; 5 Department of Perioperative Medicine, Clinical Center, National Institutes of Health, Bethesda, Maryland, United States of America; 6 Medical Virology Section, Laboratory of Infectious Diseases, National Institute of Allergy and Infectious Disease, National Institutes of Health, Bethesda, Maryland, United States of America; 7 Center for Infection and Immunity, Columbia University, New York, New York, United States of America; 8 Laboratory of Clinical Infectious Diseases, National Institute of Allergy and Infectious Disease, National Institutes of Health, Bethesda, Maryland, United States of America; Charité-University Medicine Berlin, Germany

## Abstract

Despite the important diagnostic value of evaluating antibody responses to individual human pathogens, antibody profiles against multiple infectious agents have not been used to explore health and disease mainly for technical reasons.  We hypothesized that the interplay between infection and chronic disease might be revealed by profiling antibodies against multiple agents. Here, the levels of antibodies against a panel of 13 common infectious agents were evaluated with the quantitative Luciferase Immunoprecipitation Systems (LIPS) in patients from three disease cohorts including those with pathogenic anti-interferon-γ autoantibodies (IFN-γ AAB), HIV and Sjögren’s syndrome (SjS) to determine if their antibody profiles differed from control subjects.  The IFN-γ AAB patients compared to controls demonstrated statistically higher levels of antibodies against VZV (*p*=0.0003), EBV (*p*=0.002), CMV (*p*=0.003), and C. albicans (*p*=0.03), but lower antibody levels against poliovirus (*p*=0.04). Comparison of HIV patients with blood donor controls revealed that the patients had higher levels of antibodies against CMV (*p*=0.0008), HSV-2 (*p*=0.0008), EBV (*p*=0.001), and C. albicans (*p*=0.01), but showed decreased levels of antibodies against coxsackievirus B4 (*p*=0.0008), poliovirus (*p*=0.0005),   and HHV-6B (*p*=0.002). Lastly, SjS patients had higher levels of anti-EBV antibodies (*p*=0.03), but lower antibody levels against several enteroviruses including a newly identified picornavirus, HCoSV-A (*p*=0.004), coxsackievirus B4 (*p*=0.04), and poliovirus (*p*=0.02). For the IFN-γ AAB and HIV cohorts, principal component analysis revealed unique antibody clusters that showed the potential to discriminate patients from controls.  The results suggest that antibody profiles against these and likely other common infectious agents may yield insight into the interplay between exposure to infectious agents, dysbiosis, adaptive immunity and disease activity.

## Introduction

Although there are many known pathogenic viruses and microbes (e.g. HIV, HBV and *Mycobacterium tuberculosis*) that cause human disease [1], a comprehensive catalogue of human infectious agents and their impact on health remains largely incomplete. A previous estimate placed the number of human pathogens at approximately 1400 distinct agents [2], but in the last decade many new infectious viruses and bacteria have been identified suggesting that the number is actually much higher [3]. Moreover, a major gap exists regarding our understanding of whether microbes and viruses, which are not generally recognized to cause chronic illnesses, show more complex interactions in chronic diseases. High-throughput DNA sequencing has been used as one approach to catalogue the human microbiome [3]. Several of these comprehensive DNA-based metagenomic studies have focused on the bacteria of the gut and identified microbial imbalances (called dysbiosis) in several chronic diseases including Crohn’s disease [4,5], type 2 diabetes [6,7] and obesity [8,9]. In the case of type 2 diabetes patients, an increase in the abundance of several types of pathogenic bacteria, as well as a decrease in butyrate-producing bacteria were detected [6]. While these metagenomics studies have been highly informative, broad, quantitative, host immune profiles have not been used to study host-infectious agent interactions. In the current study we focused on whether humoral profiles against multiple infectious agents were altered in several chronic diseases. 

Antibody detection is an important criterion for diagnosis of many pathogens and is important for understanding and monitoring immune responses to infectious agents and vaccines [10]. In many cases, antibody levels can vary over time and can be used to monitor pathogen clearance, dormancy, reinfection and/or reactivation. Despite the usefulness of clinical immunoassays, most current technologies do not offer the sensitivity and specificity needed to broadly assess humoral responses against multiple infectious agents on the same diagnostic platform. We have developed Luciferase Immunoprecipitation Systems (LIPS) to generate high definition antibody profiles for infection and autoimmune diagnostics and monitoring humoral responses against a variety of infectious agents [11]. LIPS is based on chimeric genes encoding target antigens that are fused to *Renilla* luciferase and expressed in mammalian cells. Crude extracts of light emitting antigens are then prepared without purification and employed in immunoprecipitation assays to quantify specific antibodies. LIPS offers a wide dynamic range of antibody detection, which can be used to distinguish different clinical conditions caused by the same infectious agent [12,13]. Due to its standard format, LIPS is an ideal technology to generate antibody profiles against multiple infectious agents in parallel and forms the basis of the current report. 

We hypothesized that antibody profiles against multiple infectious agents might be altered in chronic disease, where the immune system is compromised, reflecting the interplay between infection by these agents, host immune responses and/or disease activity. To test this hypothesis, we examined three different disease cohorts: patients with IFN-γ AAB [14], HIV infection and SjS [15]. While immunodeficiency caused by HIV infection has been extensively studied, less is known about patients with IFN-γ AAB who are immunocompromised due to autoantibodies that neutralize IFN-γ cytokine signaling activity making them particular susceptibility to severe infection by a variety of non-tuberculosis mycobacteria [14]. Sjögren’s syndrome (SjS) is a relatively common autoimmune disease characterized by immune attack on the salivary and lacrimal glands, which has been proposed to potentially have an infectious basis. Here from our study of patients and cases from three chronic diseases, we provide evidence that altered antibody profiles against common infectious agents are a frequent phenomenon in chronic immune disease and suggest that this approach might be useful for studying immune function and patient subsets in these and other diseases.

## Material and Methods

### Ethics Statement

The studies were approved by Institutional Review Boards of National Institute of Allergy and Infectious Disease or National Institute of Dental and Craniofacial Research. Informed written consent was obtained from all subjects in accordance with the human experimentation guidelines of the Department of Health and Human Services at the NIH, and the studies were conducted according to the principles expressed in the Declaration of Helsinki. 

### Patient cohorts and control subjects

To explore the interplay between infection and chronic immune disease, we studied three different disease cohorts. For a group of immunodeficient patients, an HIV cohort was chosen. The two other cohorts studied were autoimmune conditions: patients with IFN-γ AAB [14] and SjS [15]. The IFN-γ AAB cohort comprised patients (n=23) showing high levels of autoantibodies against IFN-γ (Table **S1**). All 23 IFN-γ AAB patients used in our study had the defining feature of this syndrome, infection by a variety of nontuberculous mycobacteria and 11 of the patients also had other opportunistic infections. Geographically matched blood donors from Taiwan and Thailand without autoantibodies against IFN-γ were used as controls (control group A; n=22). The characteristics of both the cases and controls are shown in Table **S1** and were randomly selected from a larger group of samples that has been previously described [14]. 

The HIV cohort contained HIV-infected patients (n=23) and healthy blood donors (n=23; control group B), which were obtained from the NIH Clinical Center, NIH, Bethesda, MD under IRB-approved protocols (Table **S2**). To minimize bias due to severe immunodeficiency, the HIV patients utilized in the study were randomly selected from a larger group of patients with relatively normal CD4 counts (mean = 530 cells/mm^3^) representing untreated and ART-treated patients. The healthy control subjects used for comparison were also randomly selected and had similar age range and gender ratio (Table **S2**).

For the SjS cohort, 23 serum samples from SjS patients and 23 blood donors (control group C) were obtained from the NIH Clinical Center, NIH, Bethesda, MD under IRB-approved protocols (Table **S3**). Due to the known heterogeneity of SjS, the patient samples selected for this study met an additional criterion of demonstrating autoantibodies against the known clinically useful SSA autoantigen (Table **S3**). Eleven of the blood donors from control group B were also used as controls in control group C.

### Infectious disease targets for LIPS serological analysis

We utilized the LIPS technology for evaluating antibody response against 13 different infectious agents including a variety of viral and fungal targets. The rationale for examining antibody responses against six different human herpesviruses (HSV-1, HSV-2, VZV, EBV, CMV and HHV-6) was because of their relative high seroprevalence in different human populations and their potential role in causing human disease, autoimmunity and their propensity for showing reactivation in immunodeficiency [16]. Similarly, the coxsackievirus B4 virus, as well as three additional enteroviruses (AstV-A1, poliovirus and Cosavirus), were examined due to their potential role in autoimmunity [17]. Antibody responses against influenza were examined as a potential general marker of humoral immunity. Finally, two fungal targets, *Pneumocystis jirovecii* and *Candida albicans*, were included in the panel because these agents cause significant infections in immunocompromised patients [18]. 

Many of the LIPS antigens used for the infectious agents described above have been previously reported along with their diagnostic performance including the gG-1 of herpes simplex virus-1 (HSV-1) [19], gG-2 of herpes simplex virus-2 (HSV-2) [19], BFRF3 (p18), BZLF2, BHRF1, and BMRF1 of Epstein-Barr virus (EBV) [20], pp165 and pp65 of Cytomegalovirus (CMV) [21], p101 of Human herpes virus-6B (HHV-6) [22] , HA2 of influenza [20], capsid of HMO-astrovirus [23] and the MSG-14 of *Pneumocystis jirovecii* [24]. New antigen constructs for additional infectious agent targets were chosen based on their known antigenicity and were generated essentially as described using the pREN2 and pREN3S vector [24]. These new constructs included the VP1 capsid protein of poliovirus (GenBank accession KC848180), the VP1 capsid protein of coxsackievirus B4 virus (GenBank accession KC848181), and enolase from *C. albicans* (GenBank accession KC848182). Antigen fusions of the glycoprotein E (gE) antigen from varicella-zoster virus (VZV) and a helicase target (GenBank accession KC848185) from a newly described picornavirus, HCoSV-A [25], were also used and will be described in detail in forthcoming manuscripts. DNA sequencing confirmed the integrity of all newly described plasmids. 

### LIPS antibody testing

A 96-well microtiter plate format of LIPS was employed to evaluate antibodies in the serum samples for each of the 13 antigen targets. For these studies, three master plates of the serum samples were constructed for each cohort for reiterative antibody testing. For generating the master plates, serum samples were diluted 1:10 in assay buffer A (20 mM Tris, pH 7.5, 150 mM NaCl, 5 mM MgCl_2_, 1% Triton X-100) in a 96-deep well microtiter plate and then stored at 4° C until use. Additional buffer blanks were also included to monitor background binding activity of the assays. As described in a detailed publication and corresponding video, LIPS testing is initiated by adding 40 μl of buffer A, 10 μl aliquots of serum (equivalent to 1 μl of serum) from the master plate, and 50 μl of each *Ruc*-antigen Cos1 cell extract typically containing an equivalent of 10^7^ light units (LU) were added to a polypropylene plate [26]. After one hour incubation at room temperature with shaking, the mixture containing IgG antibody-antigen complexes are transferred to a microtiter filter plate containing protein A/G beads for one hour additional incubation. Next the filter plate is washed with buffer on a vacuum manifold to remove unbound *Renilla* luciferase–tagged antigens. LU are then measured using a luminometer following the addition of coelenterazine substrate. All LU data were obtained from the average of at least two separate experiments and were not corrected for background protein A/G bead binding. 

### Data and statistical analysis

The antibody levels in the three cohorts were analyzed using GraphPad Prism software (San Diego, CA) and JMP (Cary, NC). Geometric mean antibody levels (GML), expressed as mean log (10) LU and 95% CI, were calculated and presented as antilog values. Unless otherwise stated, the non-parametric Mann-Whitney *U* statistical test was used for comparison of antibody titers in the control and patient groups. Since this study was exploratory, *P* values were not corrected for multiple comparisons and were rounded off to the most significant digit. For the three cohorts, the antibody responses against the 13 different infectious agents are shown from left to right corresponding to yeast, fungus, enteroviruses, influenza and herpesviruses. Correlation calculations of the antibody levels against the different infectious agents in each study were determined using the Pearson correlation analysis. The prevalence of infection of these viruses in the different cohorts was also calculated since the diagnostic performance of LIPS for several herpes viruses have been validated in previous studies [19-22] (Table **S4**). 

### Data mining

To potentially identify combinations of antibody targets useful in distinguishing patients from controls in the three studies, the RapidMiner open-source suite of data mining and machine learning software (www.rapidminer.com) was employed [27]. Principal component analysis (PCA) was used to analyze the antibody response data [28]. For the analysis, antibody responses were normalized and evaluated as a combined test to derive the corresponding principal components (PC) showing the greatest variation between all samples in each cohort. For each cohort study, PC1 and PC2 showed the highest values for describing the data. The GraphPad software was then used to graph on a biplot the PC1 vs. PC2 values for each cohort comprising all the patient and control subjects. For the IFN-γ AAB and HIV cohorts, a manually derived separation line was used to optimally separate the control subjects from patients.

## Results

### Humoral profiles in patients with high levels of neutralizing autoantibodies against interferon-γ

**Figure 1:**
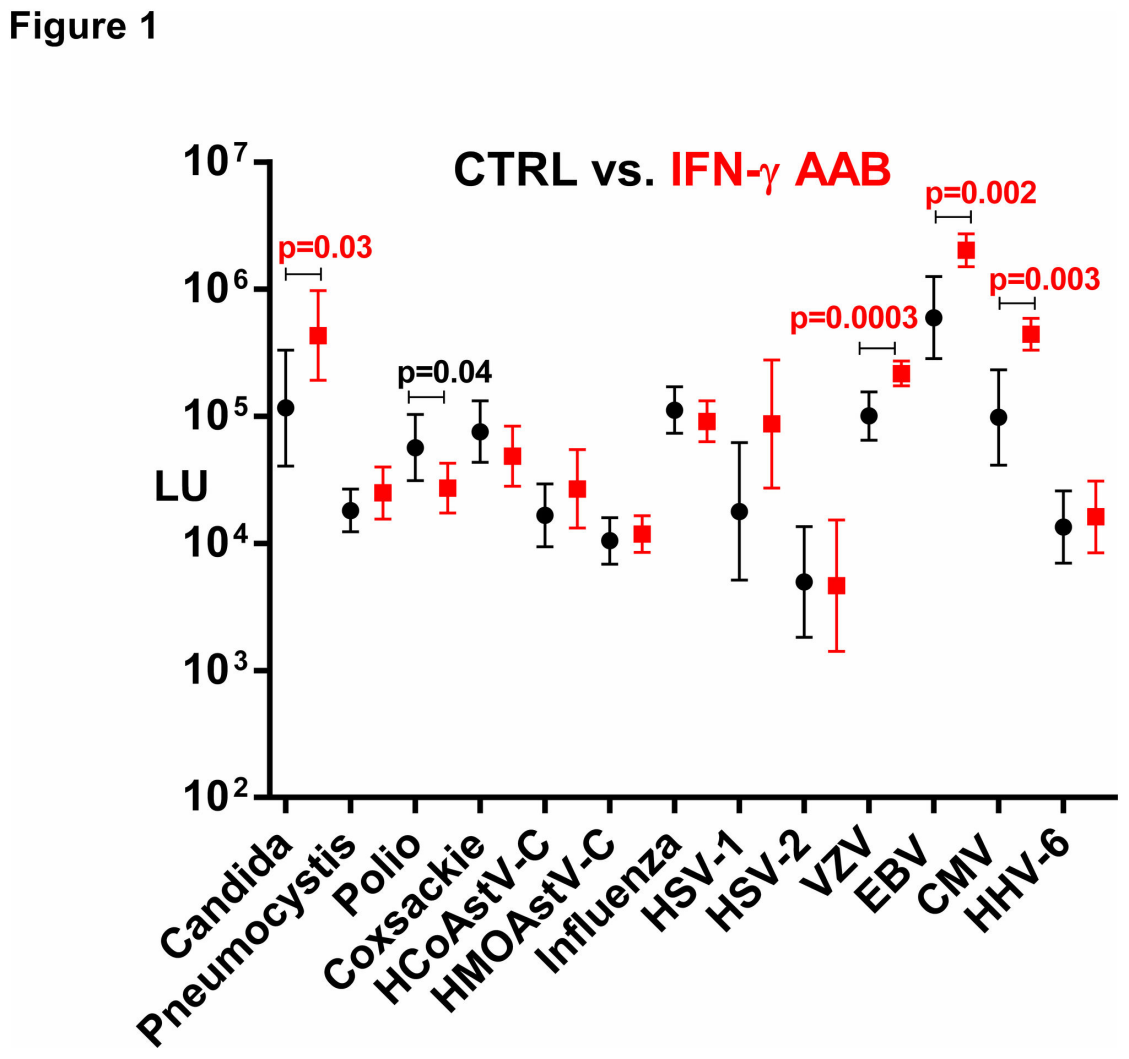
Antibody profiles against common infectious agents in IFN-γ AAB patients vs. controls. The geometric mean level and 95% CI for antibody levels against each of the 13 infectious disease targets were plotted for the 23 controls (black) and 23 IFN-γ AAB patients (red) on the Y-axis using a log_10_ scale. The different infectious agents were manually organized as described in the material and methods. Statistically significant *p* values between the two groups are shown and were calculated using the Mann-Whitney *U* test, whereby higher antibody levels in IFN-γ AAB patients are colored red, while higher levels in the controls are colored black.

Recently we described a novel immunodeficiency syndrome in a cohort of patients from Taiwan and Thailand showing unusual opportunistic infections caused by high levels of pathogenic autoantibodies against interferon-γ (IFN-γ AAB) [14]. The clinical characteristics of the IFN-γ AAB patients (n=23) along with the corresponding control subjects (control group A; n=22) are shown in Table **S1**. While the defining clinical feature of IFN-γ AAB patients is the presence of non-tuberculosis mycobacterial infection, the full spectrum of susceptibility to other infections for these patients is not known. To potentially characterize the interplay between additional infectious agents in these patients compared to controls, we employed LIPS to profile antibodies against 13 different infectious agents including viral and fungal agents. As shown in Figure 1, the geometric mean antibody levels plus 95% confidence interval for the patient and control groups against the 13 different infectious agents often showed a large dynamic range of detection often spanning 100-fold (note: Y axis is log_10_ scale). The antibody levels for many of the agents, including influenza, HCoSV-A and coxsackievirus, showed no statistical difference between the IFN-γ AAB patients and control group. However, five antigens demonstrated statistical differences between IFN-γ AAB patients and disease controls (Figure **1**). Antibodies against VZV demonstrated the greatest difference with a geometric mean level (GML) in IFN-γ AAB patients of 217,800 LU [95% confidence interval (CI); 173,400-273,500], which was significantly higher (Mann-Whitney *U* test; *p*=0.0003) than the controls with a value of 85,720 LU (95% CI; 50,140-146,500). Antibody levels against three other targets, EBV, CMV, and *Candida albicans*, also showed significantly higher values in the IFN-γ AAB patients compared to the controls with *p* values of 0.002, 0.003, and 0.03, respectively by Mann-Whitney *U* testing (Figure **1**). As shown in Table **S4**, the seroprevalence of HSV-1 infection was more common (p= 0.04) in the IFN-γ AAB patients compared to the controls, but the other seroprevalence of the four other herpesviruses (HSV-2, EBV, CMV and HHV-6) were similar between the two groups.

The IFN-γ AAB group displayed lower antibody levels in comparison to the control group only against polio vaccine (Figure **1**). The GML of antibodies against the VP1 protein of the polio vaccine in the IFN-γ AAB subjects was 27,210 LU (95% CI; 17,350-42,690) and was lower (*p*=0.04) than the controls with a value of 47,640 LU (95% CI; 24,180-93,870). Overall, these profiles demonstrate significant differences in immune response to common infectious agents in IFN-γ AAB patients in comparison to matched controls.

### Independent validation of altered anti-CMV and anti-EBV antibody profiles in patients with anti-IFN-γ autoantibodies

**Figure 2:**
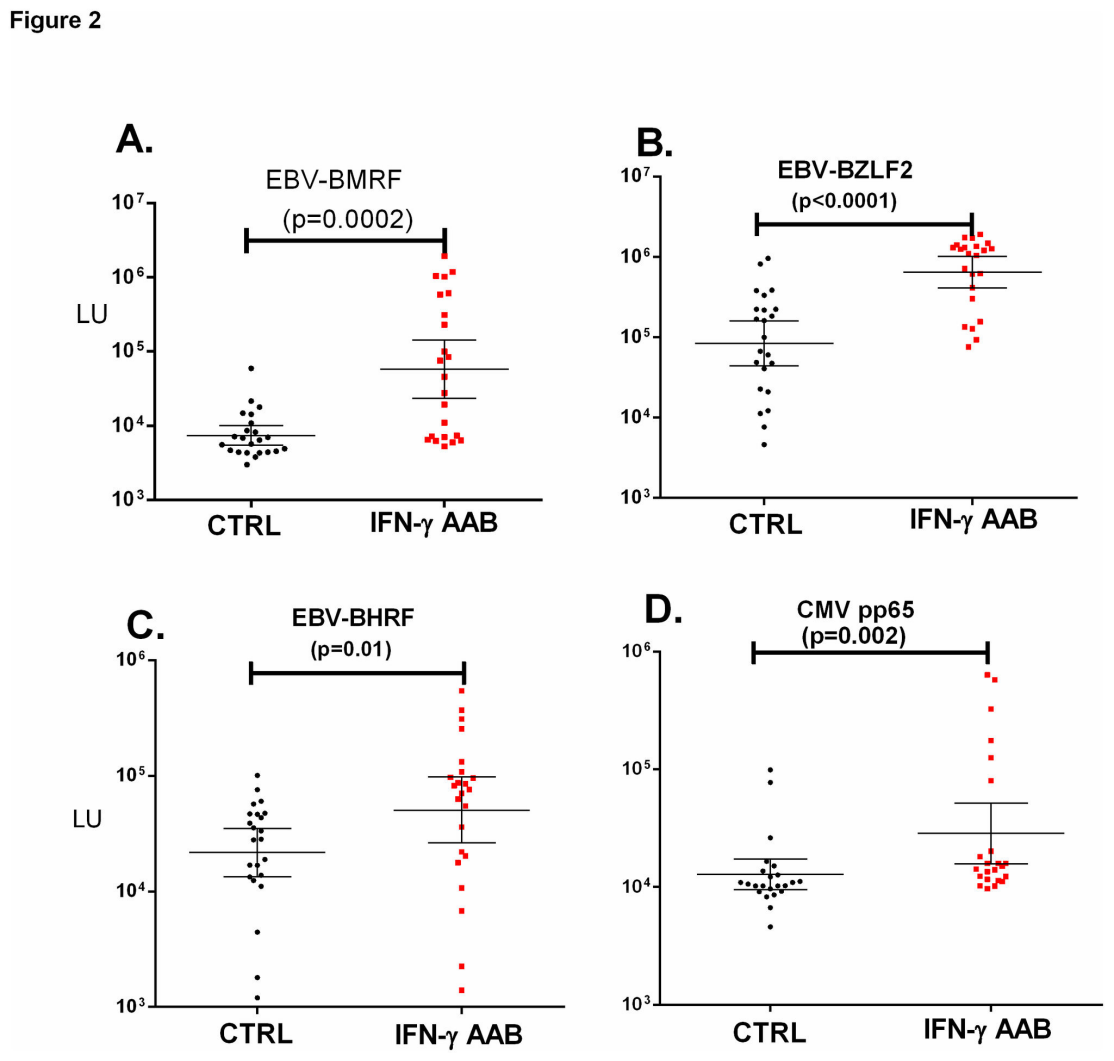
Validation of increased anti-EBV and anti-CMV antibodies in IFN-γ AAB patients. The antibody responses for (A) EBV BMRF (B) EBV BZLF2 (C) EBV BHRF and (D) CMV pp65 in the 23 controls (black) and 23 IFN-γ AAB patients (red) are plotted on the Y-axis using a log_10_ scale. Each symbol represents one individual. The geometric mean level and 95% CI for antibody levels are also shown. *P* values comparing the two groups were calculated using the Mann-Whitney *U* test.

Based on the higher antibody levels against four different herpes viruses antigenic targets in the IFN-γ AAB subjects compared to the controls, one other CMV and three additional EBV antigenic targets were evaluated in order to independently validate whether there were increased antibody levels against these two herpes viruses in the IFN-γ AAB group. Similar to the results with the BFRF3 capsid protein of EBV, all three of these lytic EBV targets, BMRF1, BZLF2, and BHRF1, showed higher levels of antibodies in the IFN-γ AAB patients compared to the controls with *p* values of 0.0002, <0.0001 and 0.01, respectively (Figure **2**). The additional CMV target, pp65, also demonstrated higher levels of antibodies in the IFN-γ AAB group compared to controls, which were statistically significant (*p*=0.002) (Figure **2**). Together these results highlight the fact that the IFN-γ AAB patients have abnormally high levels of antibodies against several herpes viruses including EBV and CMV.

### Altered antibody profiles against common infectious agent in HIV patients

**Figure 3:**
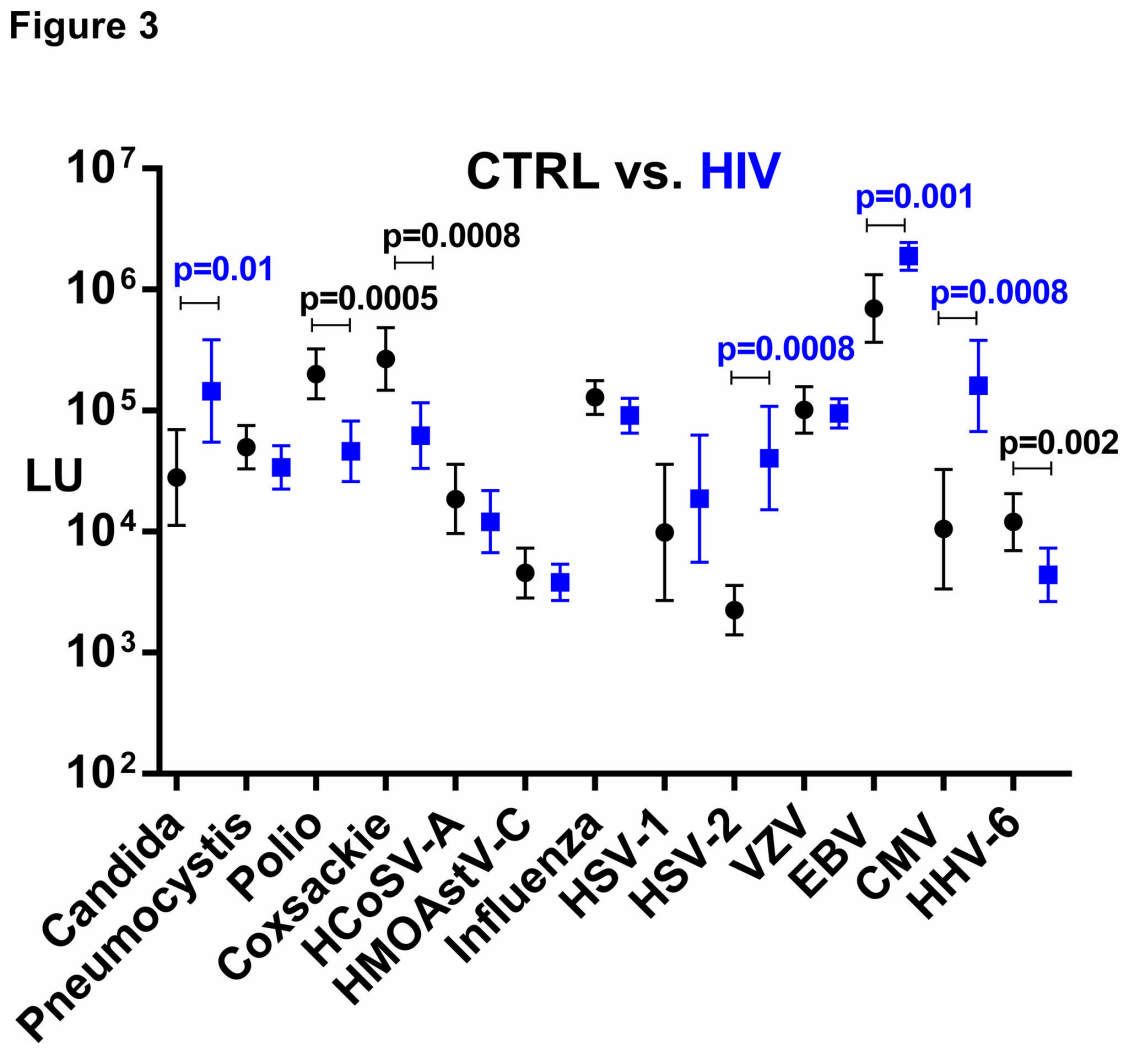
Humoral responses against common infectious agents in HIV patients vs. controls. The geometric mean level and 95% CI for antibody levels against each of the 13 infectious disease targets were plotted for the 23 controls (black) and 23 HIV patients (blue) on the Y-axis using a log_10_ scale. Statistically significant *p* values between the two groups are shown and were calculated using the Mann-Whitney *U* test, whereby higher antibody levels in HIV patients are colored blue, while higher levels in the controls are colored black.

In light of the finding that there was an altered antibody profile in the IFN-γ AAB patients, the antibody profile differences against these same infectious agents was next examined in patients with immunodeficiency caused by HIV. The clinical characteristics of the HIV patients (n=23) along with the corresponding blood donor controls (control group B; n=23) are shown in Table **S2**. Comparison of the controls with HIV subjects demonstrated that antibodies against many of the infectious agents including VZV, influenza, and HCoSV-A virus, were similar between the two groups and were not statistically different (Figure **3**). In contrast, antibody responses against four infectious agents, CMV, HSV-2, EBV, and *C. albicans*, showed significantly higher levels in the HIV subjects compared to the healthy blood donors (Figure **3**). As shown in Figure **3**, the GML of anti-CMV antibody was approximately 16-fold higher in HIV patients compared to the controls (Mann-Whitney *U* test; *p*=0.0008). However, it should be noted that there was a marked increased prevalence of anti-CMV antibodies in HIV patients (87%; 20/23) compared to healthy blood donors (43%; 10/23), thereby skewing the results (Table **S4**). Similarly, the anti-HSV-2 specific antibodies were statistically higher in the HIV-infected subjects (*p*=0.0008), and analysis of HSV-2 seropositivity revealed that the HIV subjects showed 78% seropositivity vs. the blood donors with 17% seropositivity (Table **S4**). The seroprevalence of anti-EBV antibodies in the HIV and control subjects were similar, but the GML of anti-EBV antibodies in the HIV group was approximately 18 times higher than that of the GML of the blood donor group (*p*=0.002). Lastly, the antibody levels against the *C. albicans* antigen in the HIV patients was six times higher than the control blood donor group (*p*=0.01) (Figure **3**). In comparison to the control group, HIV patients had significantly lower titers, against polio, coxsackievirus B4 and HHV6-B compared to controls with *p* values of 0.0005, 0.0008 and 0.002, respectively (Figure **3**). Analysis of the overall antibody data from the HIV patients against all 13 targets revealed that poorly correlated with each other. However, antibodies against two of the targets, poliovirus VP1 capsid and coxsackievirus B4 VP1 capsid protein weakly correlated (*R*=0.54; *p*>0.007). Protein alignment of the poliovirus and coxsackievirus B4 VP1 capsid proteins revealed 50% similarity (data not shown).

### Antibody profiles against common infectious agents in Sjögren’s syndrome patients

**Figure 4:**
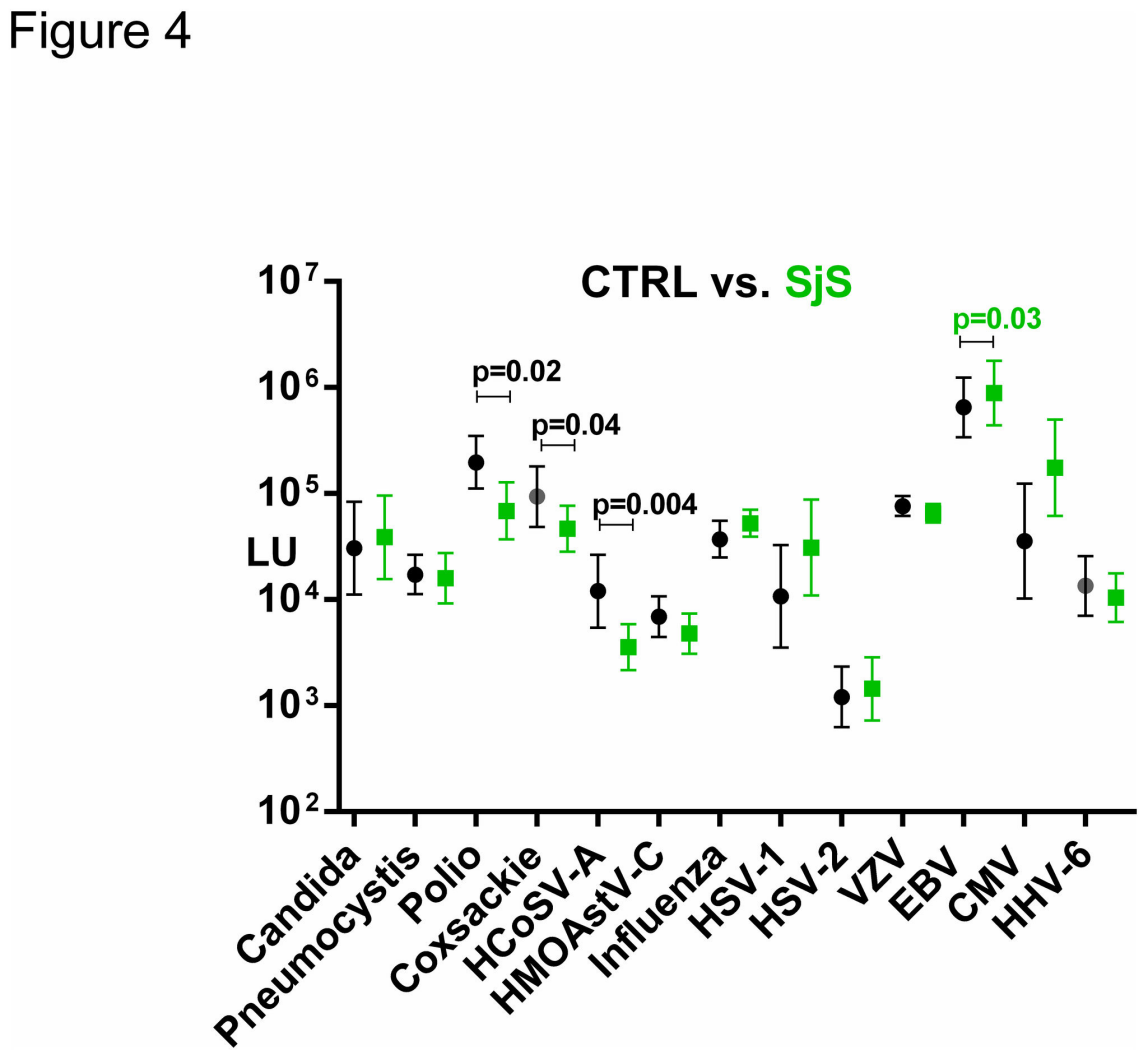
Antibody profiles against common infectious agents in SjS patients vs. **controls**. The geometric mean level and 95% CI for antibody levels against each of the 13 infectious disease targets were plotted for the 23 controls (black) and 23 SjS patients (green) on the Y-axis using a log_10_ scale. Statistically significant *p* values between the two groups are shown, whereby higher antibody levels in SjS patients are colored green, while higher levels in the controls are colored black.

Sjögren’s syndrome (SjS) is a relatively common autoimmune disease characterized by immune attack on the salivary and lacrimal glands [15]. It is not known if infectious agents play a role in the pathogenesis and symptomatology of SjS. To potentially characterize the interplay of infectious agents in the SjS cohort, we profiled antibodies against the panel of infectious agents (Table **S3**). As shown in Figure 4, comparison of blood donor controls (n=23) with SjS patients (n=23) showed that the antibody level against VZV, CMV, HSV-1, influenza and several other agents were not statistically different between the two subject groups. However, antibody levels against the EBV capsid protein showed slightly higher levels in the SjS patients compared to controls. The GML against EBV in the SjS patients was 888,000 LU (95% CI; 441,644-1,785,000) and was higher (*p*=0.03) than the controls with a value of 650,269 LU (95% CI; 340,582-1,242,000).

Besides the higher levels of antibodies against EBV in the SjS group, antibodies against three infectious agents, HCoSV-A, poliovirus, and coxsackievirus, displayed lower levels in the SjS group compared to controls (Figure **4**). The GML of antibodies against the HCoSV-A in the SjS patients showed a value of 46,280 LU (95% CI; 26,005-82,380) and was 5-fold lower (*p*=0.004) than the blood donor controls with a value of 200,784 LU (95% CI; 124,800-323,100). Antibody responses against the coxsackievirus B4 and polio also had higher GML in the controls compared to the HIV patients and were statistically significant with *P* values of 0.04 and 0.02, respectively (Figure **4**). 

### Principal component analyses reveals patient and control clusters

**Figure 5:**
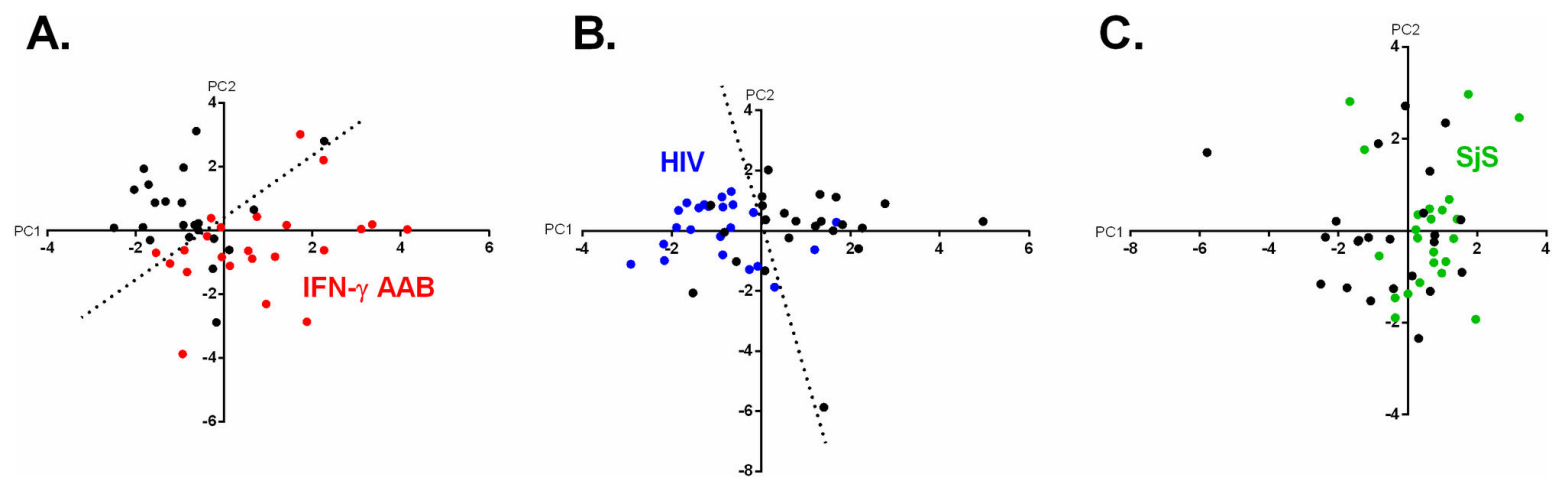
Principal component analysis of the antibody landscape reveals disease-specific clusters for the IFN-γ AAB and HIV cohorts. For each of the three studies, PCA was used separately to calculate the PC1 and PC2 values from the antibody levels for the 13 infectious agents. The PC1 vs. PC2 values for all the subjects from the (A) IFN-γ AAB (B) HIV and (C) SjS cohorts were then graphed on a biplot. In each panel, the control subjects are colored black and the IFN-γ AAB, HIV and SjS patients are colored red, blue and green, respectively. For the IFN-γ AAB and HIV cohorts, the dotted line represents a manually assigned distinction point for optimally discriminating the control subjects from patients.

One goal of these studies was to determine if the antibody landscape against common infectious agents in three diseases, in which the immune system is compromised, was different in patients vs. controls. Since our goal was to look broadly at antibody profiles, principal component analysis (PCA) was used to determine if any linear combinations of antibodies might be useful in differentiating patients from controls. The various PC values were derived for each cohort. From these analyses, the first two principal components, PC1 and PC2, in each case of the cohorts explained the largest variance of the antibody data. In the case of the IFN-γ AAB patients and controls, PC1 and PC2 explained 0.18 and 0.16, respectively of the variance from the 13 antibodies that were profiled. A biplot of PC1 vs. PC2 showed that many of IFN-γ AAB patients clustered from the controls, in which a manually derived separation line had a diagnostic performance of 86% sensitivity and 79% specificity in distinguishing cases from controls (Figure **5A**). For the HIV cohort, PC1 and PC2 explained approximately 30% of the variance and a corresponding separation line demonstrated a modest ability to differentiate HIV patients from controls with 92% sensitivity and 79% specificity (Figure **5B**). However, for the SjS cohort a similar plot of PC1 vs. PC2 was unable to clearly separate the patients from controls (Figure **5C**). Additional PCA analysis of the SjS cases and controls using the four most informative infectious agent antibody responses also had poor performance for distinguishing the two groups (data not shown). Nevertheless, the finding that the IFN-γ AAB and HIV immunodeficiency cohorts had different PCA profiles between the cases and controls highlights the complex matrix of interactions that occur between these infectious agents in these two diseases.

## Discussion

Increasing evidence suggest that the complicated repertoire and even exposure against many infectious agents, which do not cause overt disease, may influence human health and the immune system [17]. In an attempt to understand the impact of a selected group of common infectious agents, the purpose of this study was to determine whether there were altered patterns of humoral responses against a panel of 13 agents in three chronic diseases. The LIPS technology was used to systematically generate broad antibody profiles against these multiple agents. All three of the diseases studied showed significant alterations in antibody responses against single infectious agents. The multivariate PCA analysis using the cumulative antibody data for the IFN-γ AAB and HIV cohorts also showed modest ability to separate the cases from controls supporting the idea that complex interactions occur between these infectious agents in these two diseases.

In the patients with the autoimmune immunodeficiency syndrome caused by IFN-γ AAB, higher levels of antibody were observed against several different herpes viruses and *C. albicans* compared to controls. The finding that the most informative antibody response distinguishing IFN-γ AAB patients from controls was against the human herpesvirus, VZV, is consistent with the known clinical presentation of disseminated VZV infection in these patients [14]. A recent report has also documented that IFN-γ AAB patients often exhibit VZV reactivation [29]. In addition to VZV, the IFN-γ AAB patient group also showed significantly elevated antibody levels against other herpes virus members including CMV and EBV. The results showing elevated antibodies against multiple herpes viruses likely reflects the immunosuppression caused by the loss of IFN-γ and supports an important role of IFN-γ in controlling a number of different herpes virus infections. The broad antibody profiling strategy described here may also be a useful screening approach for identifying the repertoire of infectious agents that are affected by IFN-γ activity. 

From antibody profiling the HIV patients both increased and decreased antibody responses were detected against several infectious agents relative to the healthy controls. Four infectious agents, CMV, HSV-2, EBV, and *C. albicans*, elicited significantly higher levels of antibody levels in HIV patients compared to controls. The increased levels of antibodies against EBV and CMV in HIV patients are consistent with previous studies [30-33] and are likely due to the increased viral loads of these herpesviruses that are present in HIV patients [30,34]. In addition to the increased antibody responses seen in HIV patients, the HIV patients compared to controls demonstrated significantly blunted antibody responses against poliovirus, coxsackievirus and HHV-6B. While the exact reason is not known, it may reflect differences in exposure, abnormal B-cell activity and/or related immune dysfunction resulting in the loss of particular immune cell subsets. Both polio and coxsackievirus are enteric pathogens, and recent metagenomic studies have shown that there is an expansion of the enteric virome and other alterations in HIV patients [35], as well as Simian Immunodeficiency Virus-infected macaques [36]. It is also important to point out that the two immunodeficiency syndromes studied, IFN-γ AAB and HIV-infected patients, showed several differences in antibody responses. First, unlike the higher VZV antibody levels seen in IFN-γ AAB patients, no elevation of VZV antibodies was detected in HIV patients. Moreover, the decreased antibody levels in HIV patients seen against coxsackievirus B4 and HHV-6B were not observed in the IFN-γ AAB group. These subtle humoral response differences are plausible because the patients with IFN-γ AAB are only missing a single cytokine, while the HIV patients have a loss of CD4+ T-cell activity. Despite these noticeable differences, additional studies are needed to determine whether the results are disease-specific or reflect the distinct geographically-matched controls used for comparison in each of the cohorts.

In contrast to the INF-γ AAB and HIV patients, SjS patients showed elevated antibody levels against only EBV compared to controls. While several studies have implicated EBV in the pathogenesis of SjS [37-39], the antibody levels against the EBV capsid protein observed in our study were only modestly increased in the SjS group compared to controls. It is also important to point out that increased EBV antibody levels were also seen in both the IFN-γ AAB and HIV patients, suggesting the possibility that the increased EBV antibodies in SjS patients represents viral reactivation due to immunosuppression that is associated with disease pathogenesis or even treatment. The lack of significant antibody differences in SjS patients against many of the targets including CMV, *C. albicans* and HHV-6 also suggests that these agents are likely not to be directly involved in the pathogenesis of this disease. The findings of lower antibody levels against three enterovirus targets including poliovirus, coxsackievirus and HCoSV-A compared to controls was unexpected because SjS patients generally secrete high amounts of total immunoglobulin. The lower antibody levels against HCoSV-A, a new human enterovirus [25], were also not observed in the HIV or IFN-γ AAB patients. The lower antibody levels against these three enterovirus targets may reflect B-cell dysfunction, immunosuppression related to treatment, failure to produce long-lived plasma cells or altered pathogen exposure. Interestingly, a recent study has demonstrated that the gut microbiome can regulate autoimmunity via its effects on sex hormones [40]. Due to the much higher prevalence of SjS in females compared to males [15], examining antibody responses against diverse bacteria in SjS patients is an additional rich area of exploration. 

In conclusion, our results suggest that this general approach of antibody profiling many different agents could be used to more broadly identify viruses, bacteria and other organisms that might contribute to inflammation and chronic immune activation, and/or yield further insight into disease subsets and possible comorbid conditions. It is also likely that the performance of this approach could be improved by additional informative antibody targets. Since LIPS requires small amounts of serum, over 500 different agents could be profiled with approximately 1 ml of serum. This antibody profiling approach might also be useful both for exploring the pathogenesis and diagnosis of diseases that lack readily definable clinical tests such as chronic fatigue syndrome and Kawasaki disease. Future studies incorporating an even broader array of targets including against different bacteria and viruses are likely to uncover more evidence of dysbiosis in these and other diseases. 

## Supporting Information

Table S1 **Characteristics of IFN–g AAB Patients and Controls.**
(TIF)

Table S2 **Characteristics of the HIV Patients and Controls Blood Donors.**
(TIF)

Table S3 **Characteristics of the Sjögren Syndrome Patients and Controls.**
(TIF)

Table S4 **Seroprevalence of Herpesvirus Infection in the Samples.**
(TIF)
