# Supplementary figures and images for: Altered Antibody Profiles against Common Infectious Agents in Chronic Disease
Source: PLoS One. 2013 Dec 2;8(12):e81635. doi: 10.1371/journal.pone.0081635 (PMC3847058; doi:10.1371/journal.pone.0081635)

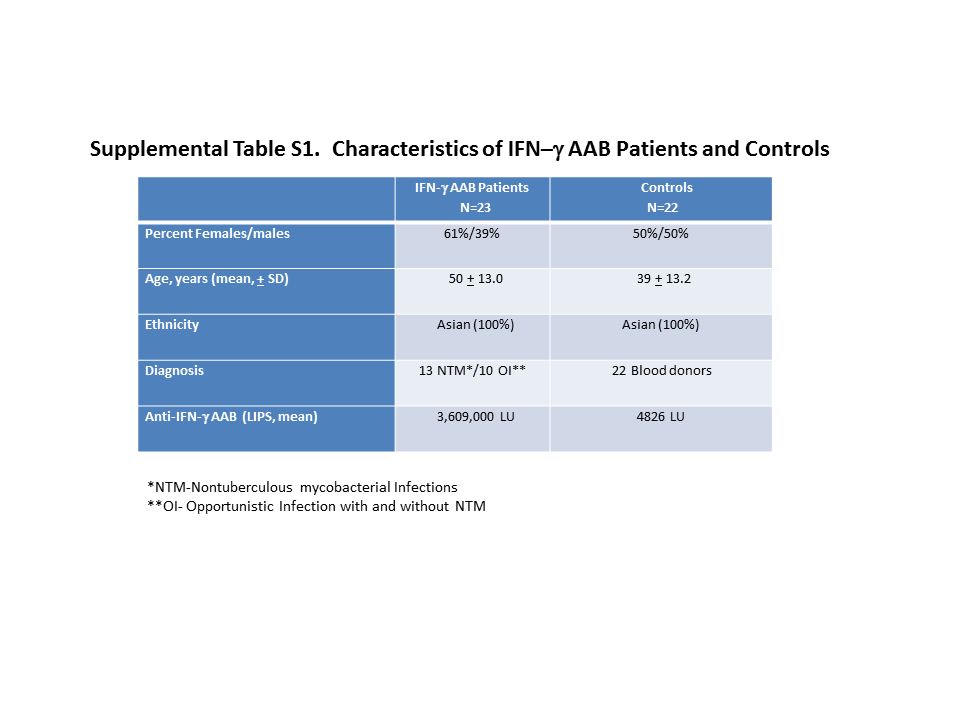

Supplement: Table S1 — Characteristics of IFN–g AAB Patients and Controls. (TIF) [file pone.0081635.s001.tif]

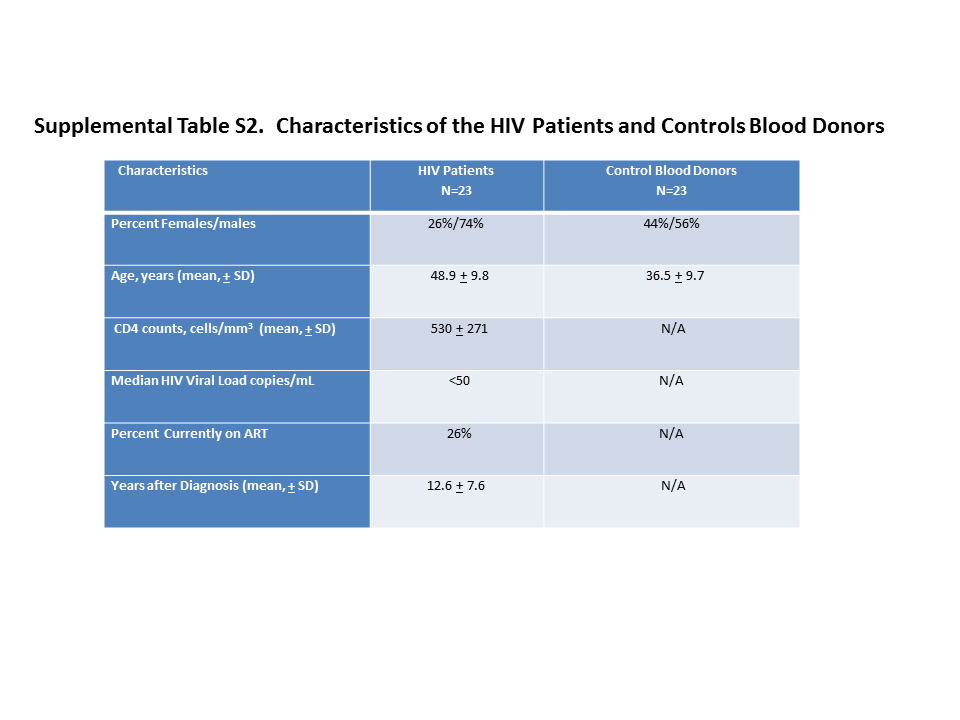

Supplement: Table S2 — Characteristics of the HIV Patients and Controls Blood Donors. (TIF) [file pone.0081635.s002.tif]

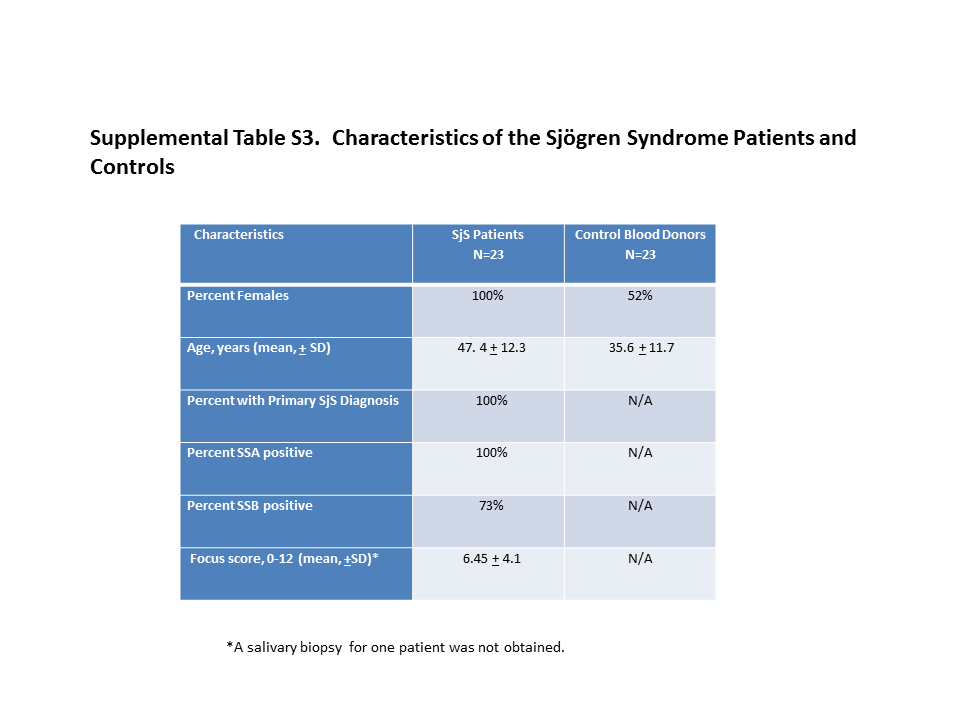

Supplement: Table S3 — Characteristics of the Sjögren Syndrome Patients and Controls. (TIF) [file pone.0081635.s003.tif]

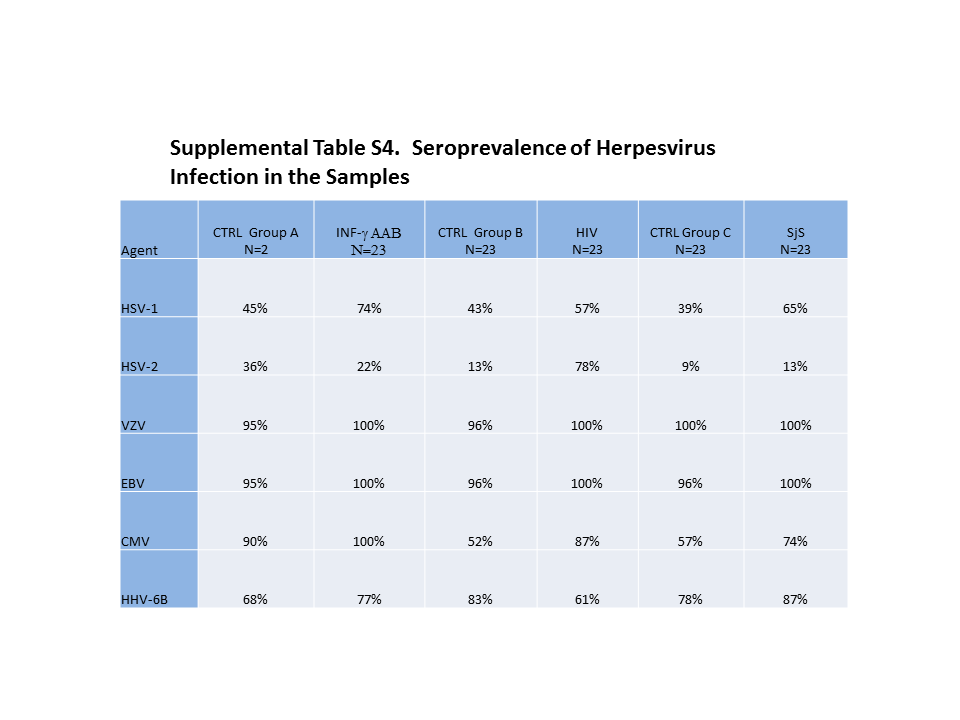

Supplement: Table S4 — Seroprevalence of Herpesvirus Infection in the Samples. (TIF) [file pone.0081635.s004.tif]
